# How to use (and not to use) movement‐based indices for quantifying foraging behaviour

**DOI:** 10.1111/2041-210X.12943

**Published:** 2017-12-18

**Authors:** Topaz Halperin, Michael Kalyuzhny, Dror Hawlena

**Affiliations:** ^1^ Department of Ecology, Evolution & Behavior Alexander Silberman Institute of Life Sciences The Hebrew University of Jerusalem Jerusalem Israel; ^2^ Herpetological Collection National Natural History Collections The Hebrew University of Jerusalem Jerusalem Israel

**Keywords:** active forager, ambush forager, animal movement analysis, behavioural indices, foraging mode, lizards, movement per minute, proportion time moving

## Abstract

1. Movement‐based indices such as moves per minute (MPM) and proportion time moving (PTM) are common methodologies to quantify foraging behaviour. We explore fundamental drawbacks of these indices that question the ways scientists have been using them and propose new solutions.
2. To do so, we combined analytical and simulation models with lizards foraging data at the individual and species levels.
3. We found that the maximal value of MPM is constrained by the minimal durations of moves and stops. As a result, foragers that rarely move and those that rarely stop are bounded to similar low MPM values. This implies that (1) MPM has very little meaning when used alone, (2) MPM and PTM are interdependent, and (3) certain areas in the MPM‐PTM plane cannot be occupied. We also found that MPM suffers from inaccuracy and imprecision.
4. We introduced a new bias correction formula for already published MPM data, and a novel index of changes per minute (CPM) that uses the frequency of changes between move and stop bouts. CPM is very similar to MPM, but does not suffer from bias. Finally, we suggested a new foraging plane of average move and average stop durations. We hope that our guidelines of how to use (and not to use) movement‐based indices will add rigor to the study of animals’ foraging behaviour.

## 1 INTRODUCTION

It was not until the late 1970s that scientists began using movement‐based indices to quantify animals’ foraging behaviour (Huey & Pianka, 2007). The first to do so were E.R. Pianka, R. B. Huey and C. Cavalier who recorded the “distance and duration of each move and duration of each stop” of seven Kalahari lizard species (Huey & Pianka, 1981, 2007). They used these data to calculate four foraging indices: moves per minute (MPM), proportion time moving (PTM), mean velocity and velocity moving (Huey & Pianka, 1981; Pianka, Huey, & Lawlor, 1979). The former two indices, MPM and PTM (for details on their calculations see Box 1), have become very popular, and are still being utilized extensively across taxa, especially in reptiles (e.g. Baeckens et al., 2017; Reilly, McBrayer, & Miles, 2007; Scales & Butler, 2015). This is mainly because such simple indices remain advantageous for comparative evolutionary‐ecological studies (e.g. Halperin, Carmel, & Hawlena, 2017; Scales & Butler, 2015), in spite of rapid methodological advancements that now enable researchers to obtain and analyse high‐resolution spatiotemporal movement data. In this paper, we expose fundamental drawbacks of MPM that seriously question the ways scientists have been using it for more than three decades, and propose guidelines to avoid these pitfalls.

The MPM is a simple, intuitive and easily measured index, which has therefore been used in hundreds of studies (e.g. *Lizards:* Reilly et al., 2007; *Fish:* Davis, Spencer, & Ottmar, 2006; Fu et al., 2009; Radabaugh, 1989; *Birds:* Botero‐Delgadillo & Bayly, 2012; McLaughlin, 1989; Newell et al., 2014; Pomara, Cooper, & Petit, 2003; *Snakes:* Hansknecht & Burghardt, 2010; *Insects:* Ferris & Rudolf, 2007; Mundahl & Mundahl, 2015). For example, to date, the foraging behaviour of 167 lizard species was characterized by MPM, occasionally as the sole foraging index, but more often coupled with PTM or other less common foraging indices (e.g. average duration of movement (AD)—Cooper, 2005a; proportion of predation attacks initiated while moving (PAM)—Cooper & Whiting, 1999; mean velocity (MV)—Huey & Pianka, 1981). Researchers have used MPM to compare foraging behaviour across species (Reilly et al., 2007), explore questions regarding the foraging mode controversy (i.e. whether foraging behaviour has two discrete modes‐ Butler, 2005; Cooper, 2005b), and search for association between foraging behaviour and other variables, such as morphology (Botero‐Delgadillo & Bayly, 2012), physiology and performance (Miles, Losos, & Irschick, 2007; Verwaijen & Van Damme, 2008b), colouration (Halperin et al., 2017; Hawlena, 2009; Hawlena, Boochnik, Abramsky, & Bouskila, 2006), and environmental conditions (Verwaijen & Van Damme, 2008a). Yet, the simplicity of MPM that makes it so popular also harbours intrinsic methodological problems that thus far have passed largely unnoticed.

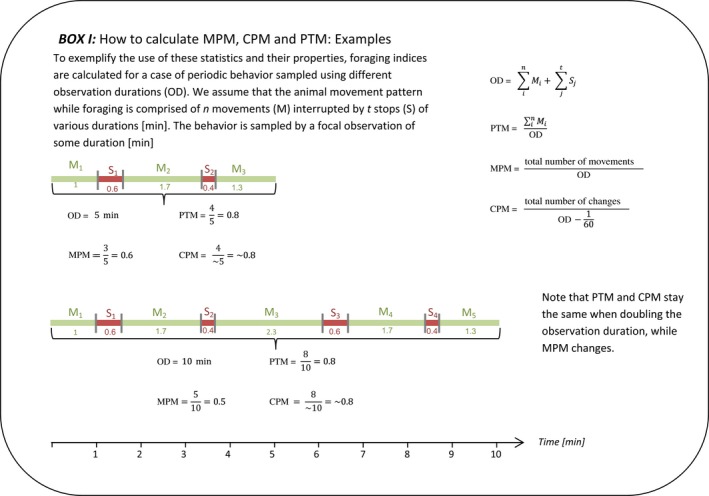

## 2 INTRINSIC LIMITATIONS OF MPM

### 2.1 The range of MPM values is determined by PTM

**Figure 1:**
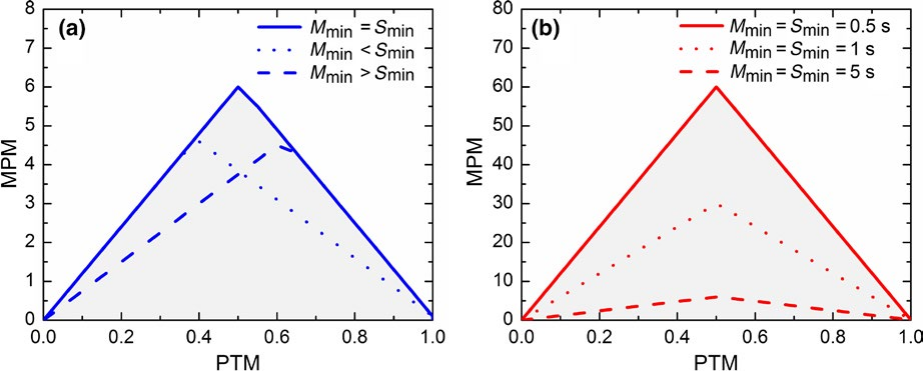
The shape and size of the triangle of possible MPM values on the MPM‐PTM plane is determined by (a) the ratio between the minimal durations of movements (*M*_min_) and stops (*S*_min_), and (b) by their minimal values. When *M*_min_ and *S*_min_ are equal then the possible MPM values are limited within the shape of an isosceles triangle. Yet, when there is a difference between *M*_min_ and *S*_min_ this triangle becomes asymmetric. Larger *M*_min_ and *S*_min_ values lead to smaller angles at the base of the triangle, and therefore to smaller range of possible MPM values

The first and most fundamental drawback of MPM is that sit‐and‐wait animals that rarely move and active foragers that rarely stop can have similar low MPM values. In fact, the maximal value of MPM for a given foraging behaviour is constrained by the minimal duration of moves, *M*_min_, and stops, *S*_min_. For low values of PTM, the value of MPM is inherently limited by *M*_min_, and its maximal value is: MPM_max_ = PTM/*M*_min_, where PTM is a fraction and *M*_min_ is in minutes. This is because only a few discrete movements can be conducted in a short period of time. For example, if PTM is 0.1, and *M*_min_ is 1/60 then MPM cannot exceed 6. For high values of PTM, animals move almost continuously, and therefore the number of distinct moves is limited by the minimum stop duration *S*_min_: MPM_max_ = [(1−*PTM*)/*S*_min_ + 1]/OD, where OD is observation duration. Intermittent PTM values permit higher MPM_max_ values. Thus, the range of possible MPM values when plotted on a MPM‐PTM plane is bounded within limits shaped like a triangle (with staircase lines edges). As shown above and elaborated in Figure 1, the shape and size of this triangle‐like area of possible MPM values is determined by the minimal durations of movements and stops. Please note that the above formulas are simplified approximations under the assumptions that *M*_min_ and *S*_min_ are of similar time‐scale, and also much shorter than the OD. These assumptions are met in all of the behavioural studies we examined. The exact formulas are derived in Appendix I.

**Figure 2:**
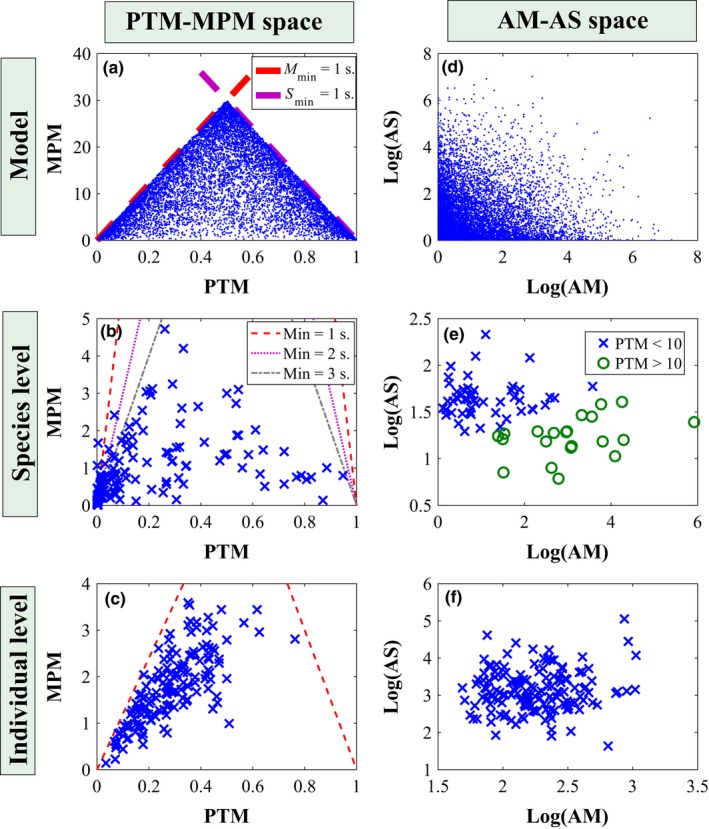
Comparison of analyses using PTM‐MPM space and AM‐AS space. The modelled data (a,c) were obtained using 10^2^ simulations, 2,400 time steps each. The species‐level data (b,d) were obtained using a literature survey of 162 species for the PTM‐MPM and 79 species for AM‐AS, and considering several possible minimal stop and move durations. Individual‐level data (c,f) presents an analysis of 155 raw movement sequence data, where *M*_min_ = 5 and *S*_min_ = 4. Note that the empirical MPM values (b,c) are very close to the MPM_max_, indicating they may be seriously affected by this constraint

To explore these limitations and their relevance to realistic scenarios we ran simulation models (Figure 2a), analysed published MPM and PTM data of 162 lizard species (Figure 2b), and examined our own foraging sequences of 155 individual lizards that belong to seven lacertid species (Figure 2c, see appendix II for elaboration). We found that, indeed, PTM‐MPM values are confined by triangle‐like limits. Moreover, using a realistic range of *M*_min_ and *S*_min_ values to establish the triangle‐like MPM_max_ limits (1–3 s; Figure 2b), we noticed that a considerable number of the published MPM values approached these limits. This was especially true for MPM values that were associated with low PTM values. Our findings indicate that many of the published MPM values are indeed constrained by their MPM_max_. The same pattern was clearly evident also in the individual‐level data, where *M*_min_ and *S*_min_ are known (Figure 2c).

As mentioned above, the constraint on MPM is determined by *M*_min_ or *S*_min_. These values are defined either arbitrarily, or due to technical difficulties to record very short moves or stops. Thus far, only very few studies have explicitly reported the exact *M*_min_ and *S*_min_ values used (e.g. Hawlena et al., 2006), most likely due to the lack of awareness of their critical importance. Consequently, we could not assess the actual variation of *M*_min_ and *S*_min_ values in the literature. Using different *M*_min_ and *S*_min_ values to characterize the exact same data series may yield very different MPM and MPM_max_ estimates. This is because as *M*_min_ decreases more movement bouts are included in the calculation, hence MPM increases. Thus, the inconsistent determination of *M*_min_ and *S*_min_ both within and between studies may bear severe implications to the ways this popular index is being used and interpreted.

It is important to note that the determination of *M*_min_ and *S*_min_ does not reflect just methodological constraints that can be completely eliminated using modern technologies, such as high‐speed imaging. Instead, functional biological limitations regulated by the animal physiological and biomechanical performances are expected to define the biologically relevant values of *M*_min_ and *S*_min_ (see Kramer & McLaughlin, 2001 for possible considerations). Existing approaches for splitting behaviour into bouts can help in identifying biologically relevant and statistically sound criteria for determining *M*_min_ and *S*_min_ (Sibly, Nott, & Fletcher, 1990; Yeates, Tolkamp, Allcroft, & Kyriazakis, 2001).

### 2.2 MPM is inherently biased

Moves per minute suffers from another methodological drawback of intrinsic inaccuracy due to the fact that movements have a continuous duration, but the number of movements is discrete. That is to say, the number of discrete movements counted in a given observation may include just fractions of movement bouts, at the beginning and end of the observation. This leads to MPM values that never converge to the true movement frequency, regardless of the sample size. To clarify this issue, let us consider a species that has a distinct movement pattern of two brief stops during 5 min (as in Box 1). This means that in 5 min this species conducts two short breaks and three movements; in 10 min it conducts four breaks and five movements; in 20 min—eight breaks and nine movements, etc. Therefore, the estimate of MPM, $\hat{\text{MPM}}$, of the 5‐min observations (0.6) neither equals to that of the 10‐min (0.5) nor to that of the 20‐min observations (0.45) (See Figure S1 for numerical example of $\hat{\text{MPM}}$ causes of bias).

**Figure 3:**
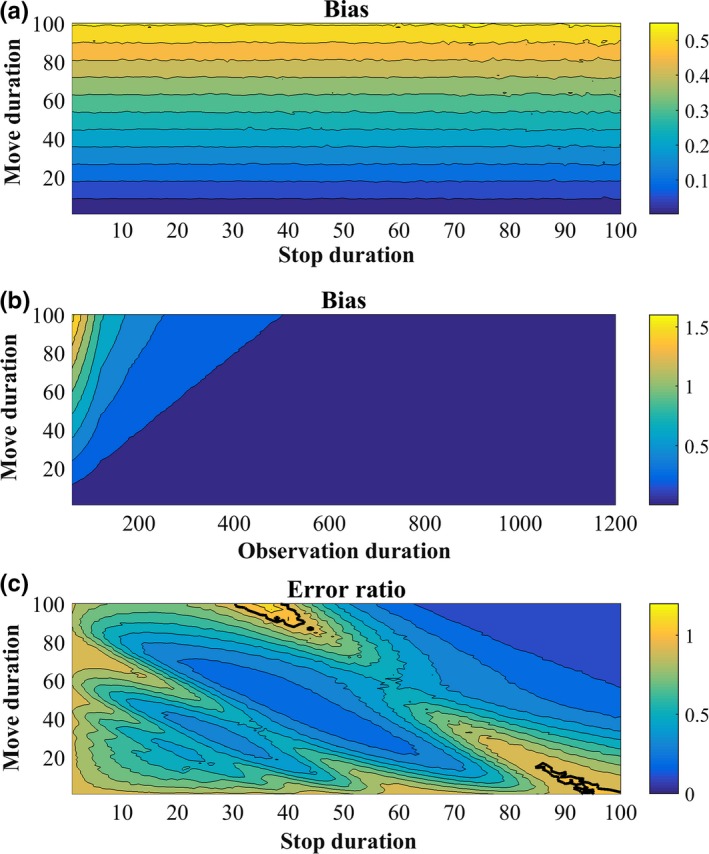
Theoretical analysis of bias and error of movement frequency indices using simulations. In (a), the relative bias of MPM (Equation (1)) is presented against the move and stop duration parameters, *M* and *S*, of the simulation model for 3‐min observations. In (b), we use *S* = 20 and study the effect of *M* and OD on relative bias. In (c), we calculated the ratio of the variance of CPM/2 to the variance of MPM. The bold black line is the contour 1, above which MPM has lower variance, which rarely happens. For each parameter combination we generated a long movement sequence of 2·10^6^ s and calculated its MPM, then sampled from it 10^4^ short sequences and calculated their MPM. The variance of these MPMs was used as the error in (c) and their mean was compared to the MPM of the long sequence to determine bias in (a) and (b)

Deriving a model for the bias, we found that the relative bias in MPM is given by the formula:

(1) $${\text{Bias}}_{\mathrm{rel}}=\frac{E\left(\hat{\text{MPM}}-\text{MPM}\right)}{\text{MPM}}=\frac{\text{AM}}{\text{OD}},$$

where AM is the average move duration and OD is the observation duration. Please consult appendix III for detailed explanations and derivations of this expression, and Figure 3a,b for simulations confirming it. This formula indicates that the relative bias is independent of the average stop duration (AS) and linearly dependent on AM and on 1/OD. Hence, the bias will be considerable for short observations of animals with long move durations.

**Figure 4:**
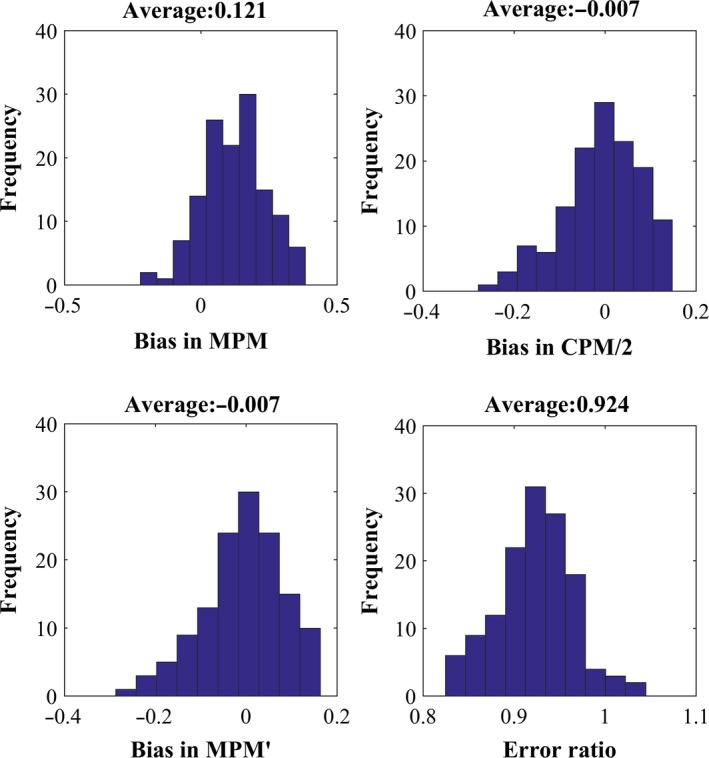
Empirical analysis of bias and error of movement frequency indices. For each of the 134 long (>20 min.) lizard movement sequences we sampled ~1,000 two min. sequences starting at 1‐s intervals. Then, for each of the 134 sequences we calculated the mean difference in (a) MPM (b), MPM′ and (c) CPM/2 between the short samples and the original long sequence as an approximation of the bias for that sequence. Histograms of these bias estimates are presented. In (d) we calculated the ratio between the variances in the estimates of CPM/2 and MPM of the short samples. The histogram of these ratios for the 134 sequences is shown. Note that CPM/2 is unbiased and has lower variance than MPM, and MPM′ is also unbiased with roughly the same distribution

To test whether this bias indeed occurs in real foraging data, we used our records of the movement sequences of lacertid lizards. We calculated the MPM of the 134 individual activity sequences with an OD of at least 20 min. Then, for each observation we sampled all short sequences of 2 min, starting at 1‐s intervals. This resulted in a little more than 1,000 2‐min samples per observation, depending on the exact duration of the original observation. The mean MPM of these short samples was compared to the MPM of the entire sequence, as an approximate estimate of the bias for an individual (Figure S2). We found positive bias for 85% of the individuals, with a mean bias of 0.121 over all 134 lizards (Figure 4a). This is likely an underestimate, since the 20‐min sequences do not really represent unbiased infinitely long activity sequences.

The absolute bias equals PTM/OD, where PTM is a fraction and OD is in minutes (appendix III eq. 4). Indeed, a regression of the bias against PTM (where each data point is the bias in estimation for one individual) detected a linear relationship with slope 0.55 ± 0.115 CI (at α = 0.05, *r* = .63; *p* = .379), statistically indistinguishable from the expected slope of 0.5 for OD = 2 min. This analysis further corroborates our bias model.

Reported MPM values are expected to suffer not just from intrinsic bias but also from imprecision. As mentioned above, the inherent MPM bias largely depends on the OD. Yet, OD may differ substantially between studies (Perry, 2007; see appendix IV). Moreover, in many studies, researchers have pooled observations of different durations to characterize the foraging behaviour of a single species, and reported only the minimal observation duration or the average duration (e.g., Cooper, Vitt, Caldwell, & Fox, 2001; Huey & Pianka, 1981; Sales & Freire, 2015). Pooling together MPM estimations that are based on various ODs, hence including different biases, may increase the errors in the estimation of summary statistics such as species‐average MPM. Together, the problems of inaccuracy and imprecision add yet another question mark to the validity of MPM as a reliable index for foraging behaviour.

## 3 IMPLICATIONS OF MPM DRAWBACKS TO DATA INTERPRETATION

We now explore how the methodological drawbacks of MPM may affect data analyses and the interpretation of results. First, when used as a stand‐alone index, similar MPM values can reflect substantially different foraging behaviours. For example, an ambush forager that hardly moves and a widely foraging species that barely stops can both have very low MPM values. This strongly suggests that the theoretical cut‐off values of MPM = 1, which is sometimes used to separate foraging modalities (e.g. Butler, 2005; Randrianantoandro & Hobinjatovo, 2011; Scales & Butler, 2015; Scales, King, & Butler, 2009) is fundamentally flawed. Indeed, Cooper (2005b) found in a cluster analysis that was conducted on lizard MPM values that species with markedly different behaviours were grouped together. Thus, conclusions founded solely on MPM (e.g. McLaughlin, 1989) or on associations between MPM and other variables often bear little biological meaning and should be treated with extra caution.

Moves per minute is often used complementarily with PTM to reveal variation in foraging behaviours that cannot be identified using PTM alone (Cooper, 2005b; Perry, 2007; Perry et al., 1990). An acknowledged limitation of PTM is that animals can have identical PTM values while using very different foraging behaviours (Perry et al., 1990). For example, in a 10‐min observation, an animal that moves continuously in a one movement bout of 4 min, and an animal that moves in 16 short bouts of 0.25 min will both have PTM values of 0.4. On the other hand, the MPM values of these hypothetical animals are 0.1 and 1.6 respectively. Thus, using the MPM‐PTM foraging plane can reveal the behavioural differences between the two animals. Yet, as we clearly demonstrated, the MPM‐PTM plane is bounded within triangle‐shaped limits. Therefore, any correlation found between PTM and MPM (as in Cooper, 2005b) may reflect the dependency between these metrics, caused by mere methodological constraints. As a result, studies that examine correlates of MPM and PTM (Cooper, 2007; Cooper et al., 2001; Miles et al., 2007; Perry et al., 1990; Verwaijen & Van Damme, 2007) may suffer from collinearity. In addition, studies that examine correlation between MPM and PTM may reach contrasting results just because they included different ranges of PTM values (i.e. positive correlation: Cooper et al., 2013; no correlation: Cooper, 2005b).

**Figure 5:**
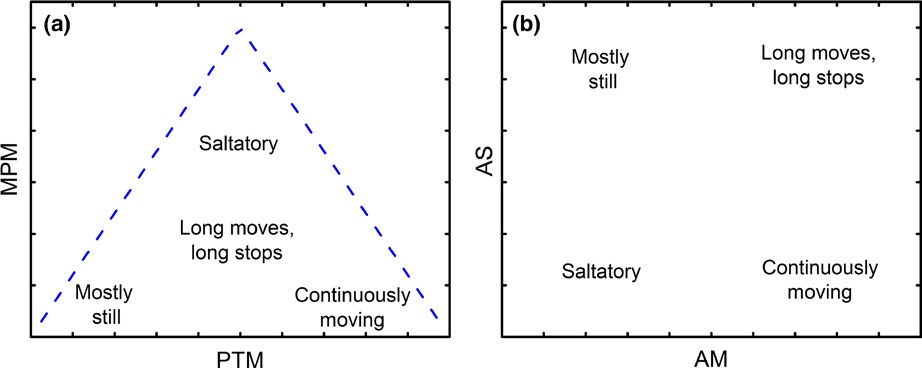
Relationships between different parts of the (a) PTM‐MPM space and (b) the AM‐AS space

The triangle‐like constraint on the MPM‐PTM plane may further undermine the way scientists have used it to study foraging behaviour. Specifically, certain combinations of MPM and PTM, such as those predicted by Fig. 1 in Butler (2005) (“Short Spurts” and “Stop‐and‐go”) or Fig. 1 in Cooper (2005b) (“frequent, very brief movements relative to pauses” and “frequent, brief movement‐ briefer pauses”), are not possible (Figure 5). This means that the absence of species from these areas of the MPM‐PTM plane does not reflect data deficiency (as was previously suggested, see Cooper, 2005b), or selective pressures against these strategies, but rather it stems from an intrinsic methodological limitation.

As we show in Appendix III, the bias in MPM estimations is particularly substantial for short observation of animals with long move durations (Figure 3). Many studies have used observation durations that were shorter than 5 min, at times as short as 1 min. For example, our thorough literature review of lizards’ foraging behaviour revealed that in 83 of 118 studies for which minimal OD was reported, OD_min_ was equal or smaller than 3 min (Median OD_min_ = 2.18; Appendix IV). Perry (2007) found that shorter observations often produce relatively high values of MPM and greater variability than longer observations. He explained this finding by claiming that short observations only sample part of the behavioural repertoire of the animal, and suggested conducting longer observations, especially for species with intermittent locomotion. We add that higher MPM values in shorter observations could result purely from the way the index is being calculated, and that this problem may be relevant especially for highly active species (see Figure 3).

## 4 RESOLVING A FEW DRAWBACKS OF MPM

### 4.1 Bias‐corrected estimator for MPM

Our second goal was to suggest ways to resolve some of the above‐mentioned problems of MPM. As we already mentioned, MPM estimates already exist for hundreds of species. Thus, an important challenge was to develop a tool to correct the intrinsic bias of the naïve $\hat{\text{MPM}}$ estimator. We suggest using the correction

(2) $$\text{MPM}{}^{\prime }=\hat{\text{MPM}}-\frac{\text{PTM}}{\text{OD}},$$

where MPM′ is the bias‐corrected estimator for MPM (see Appendix III for details). This correction is feasible in all cases in which the reported information includes observation duration and PTM values. To test the validity of using ${\text{MPM}}^{\prime }$ to correct the bias in $\hat{\text{MPM}}$ estimations of lizards’ foraging sequences, we applied the correction to the $\hat{\text{MPM}}$s of the 2‐min samples of the 134 long movement sequences (see above). Figure 4b shows that this correction eliminated the bias (mean bias = −0.007), demonstrating that this novel method can be very useful in correcting already published results.

The derivation of the bias‐corrected estimator MPM′ requires data on $\hat{\text{MPM}}$, PTM and OD for every individual sample. This information is quite rare in the published literature. Most reported data include only the (arithmetic) average of $\hat{\text{MPM}}$, PTM and OD. Sometimes only the total observation duration for the entire study is published. In these cases, it is still possible to apply the correction by using the mean OD (if only total observation time is available, one can divide the total duration by the number of observations), mean $\hat{\text{MPM}}$ and mean PTM values in Equation (2).

Importantly, we found that the correction using average values, while perhaps not correcting all the bias of MPM, never creates extra bias. Concisely, this is because the full bias correction MPM′ (Equation (2)), when averaged over individuals, involves reducing a number proportional to the reciprocal of the harmonic mean, while the feasible correction involved reducing a number proportional to the reciprocal of the arithmetic mean. Since the harmonic mean is never larger than the arithmetic mean, this correction factor will always correct some (or all) of the bias, but never create extra bias. Our numerical simulations confirmed this result (Appendix III). We can, therefore, recommend using the MPM′ correction even when only study‐level averages are available.

We applied the MPM′ correction to all published studies on lizards’ foraging behaviour from which we were able to extract the relevant data (see Appendix IV for details). While in many cases MPM′ differs from MPM in <1% (as we expected, since most lizard species for which MPM, PTM and OD are currently available are sit‐and‐wait foragers), in 9 of 98 studies the difference is >10% and in one study the difference is 38%. The corrected MPM′ values for all published studies on lizards’ foraging behaviour are provided in appendix IV. We encourage future comparative studies on lizards’ foraging behaviour to use these MPM′ values rather than MPM values (Appendix IV).

Our MPM bias correction relies on several assumptions, and particularly that there is no correlation between the duration of a move or stop and other moves and stops. Thus, we recommend using this expression only to correct published results for which the movements’ sequence data at the individual level are not available. If raw data on moves and stops are available, we suggest using a different estimator that overcomes the problem of splitting movement and stop bouts altogether—the number of movement‐stop or stop‐move changes per minute (CPM).

### 4.2 A new movement frequency index—CPM

Changes per minute is calculated by dividing the number of observed changes between move and stop bouts by observation duration minus 1 s (or other minimal time unit to which the observations were discretized), as no change can be observed in that last time unit of the observation (Box 1). Since half the changes are movement initiations, and since these changes are instantaneous and therefore cannot be partially sampled, CPM divided by two is an unbiased estimator of the true MPM an animal performs in a long sequence of movement. This was confirmed by exploring the bias in simulated movement sequences (result not shown) and in the 2‐min sampling of real foraging sequences of individual lizards (Figure 4c). Furthermore, our simulations (Figure 3c) and the analysis of the 2‐min samples (Figure 4d) revealed that CPM/2 also has lower variance compared to MPM. Since CPM has small bias (it is unbiased compared to a long sequence of movement) and low error, it is a superior statistic to MPM that provides similar information and maintains the original simplicity of this index.

### 4.3 AM‐AS plane

While MPM′ and CPM seem to resolve the intrinsic inaccuracy of MPM, they cannot resolve the inherent triangle‐like limitations of the MPM‐PTM plane, and their implications for subsequent analyses. Thus, we suggest using a plane of average move duration (AM) and average stop duration (AS) as an alternative approach. Cooper (2005a) examined AM (i.e. AD in the original paper) as an additional foraging index to MPM or PTM. We, instead, focus on the strengths and weaknesses of using the AM‐AS foraging plane. Figure 2 illustrates the correspondence between the MPM‐PTM and AM‐AS planes using simulated, species level (based on Cooper, 2005a) and individual foraging data. It is important to note that the AM (calculated by Cooper, 2005a) and AS values at the species levels were calculated using reported averages of MPM, PTM and OD. Consequently, these rough estimations of AM and AS may suffer from inflated inaccuracy and imprecision that may render biological analyses that use them invalid. Nonetheless, we decided that with no other data in hand, these species‐level estimations can still be valuable for exploring the pros and cons of the AM‐AS plane. First, as opposed to MPM‐PTM, the AM and AS axes are methodologically independent. Hence, foraging strategies can be assigned to any part of the plane, as depicted in Figure 5. Thus, any correlation found between AM and AS may reflect meaningful biological information. We think that the AM‐AS plane is favourable because AM and AS are easy to interpret, and do not suffer from inherent constraints or intrinsic biases. This analytical approach is useful especially for exploring evolutionary‐ecological aspects of movement behaviour across related taxa by methods of cluster analyses.

Despite these advantages, we want to emphasize three limitations of the AM‐AS plane approach. First, there is no single axis that defines foraging strategies and is capable of replacing PTM. Species‐level data in Figure 2 show that while sit‐and‐wait and active foragers (defined as below and above PTM of 10%, respectively, as in Cooper et al., 2001) are distinct on the AM‐AS plain, neither of these variables alone separates them and can be used as a stand‐alone index in comparative analyses. Second, since the number of moves and stops is usually not very large even for active foragers, and since it is necessary to drop the edges of the sampling sequence (because the entire duration of these first/last move/stop is not sampled), sample size for these variables is smaller than for PTM, which considers every unit of sampled time. Third, as opposed to PTM and MPM, the AM‐AS plane approach uses absolute values rather than standardized values. Thus, this approach may impede comparisons between unrelated taxa that differ in body size or the environment they inhabit.

## 5 GUIDELINES FOR FUTURE USE OF MPM

To encourage better use of MPM in future behavioural studies, we provide guidelines of how to use (and not to use) this index.

1. MPM should not be used in comparative studies as a stand‐alone index or to be correlated with other variables. Only in cases in which animals have similar PTM values, MPM‐like measurements can be used to uncover differences in foraging behaviour.
2. To keep MPM‐like measurements more accurate, reproducible and comparable across studies, we urge researchers to: (1) use the MPM′ correction we introduced for published MPM values, and (2) use the new CPM/2 index instead of MPM and MPM′ when raw data on moves and stops are available. Previously published results using MPM or MPM′ can easily be compared with new results using CPM/2.
3. To explore ecological‐evolutionary correlates of movement behaviour, we recommend using the AM‐AS plane rather than the MPM‐PTM plane. Only when standardized data are required to compare species should the CPM‐PTM plane be used. But in these cases, the inherent triangle‐like limitations and their implications for subsequent analyses should be considered.
4. We encourage researchers to adjust their observation protocols to address the concerns we raised. It is very important to reduce the observed *M*_min_ and *S*_min_ as close as possible to the minimal biological values that are relevant for the focal study. This can be achieved by using high‐speed imaging data and event logging software. The observation duration should include sufficient number of movement bouts and represent the natural variation in foraging behaviour. To determine the minimal representative sampling effort one can use a method similar to the collector's curve technique (Dias, Rangel‐Negrín, Coyohua‐Fuentes, & Canales‐Espinosa, 2009; Hawlena et al., 2006).
5. Last, the values of *M*_min_ and *S*_min_ as determined by the observer along with the exact OD should always be reported.

## 6 CONCLUDING REMARKS

Movement‐based indices, such as MPM and PTM, are simple, intuitive and easy to measure. Thus, these indices have been used extensively to depict and study foraging behaviour across species and systems. Yet, MPM suffers from major drawbacks that must be acknowledged to prevent misuse. We demonstrated that MPM values are constrained by the minimal move and stop durations, leading to similar low values for both active and sit‐and‐wait foragers. Also, we showed that this index suffers from intrinsic inaccuracy and imprecision. To assist avoiding these pitfalls, we developed a new bias correction formula for already published MPM data. When raw data on moves and stops are available, we proposed using a novel index of changes per minute (CPM) that is very similar to MPM, but does not suffer from bias and inflated error. It is important to note that MPM′ and CPM are similar in their interpretation and converge to the same value as MPM when the observation duration increases (up to the bias of MPM). Hence, previously published results using MPM (corrected using MPM’ when possible) can be easily compared with new results using CPM/2. We also suggested a new foraging plane of average move and average stop durations that resolves some of the inherent limitations of the MPM‐PTM plane. We want to emphasize that our goal is certainly not undermining the use of frequency‐based indices to study foraging behaviour. On the contrary, we believe that such simple and comparable movement‐based indices are still very useful to explore ecological and evolutionary aspects of foraging behaviours, especially in comparative studies. We hope that our work will add rigor to these attempts by assisting researchers to avoid common methodological pitfalls that can seriously affect further development of this important field.

## AUTHORS’ CONTRIBUTIONS

All authors conceived and developed the ideas, collected and analysed the data, and jointly wrote the manuscript. All authors contributed critically to the drafts and gave final approval for publication.

## DATA ACCESSIBILITY

Data of the lizards’ foraging behaviour indices PTM and MPM, and our new MPM’ corrections are deposited in Dryad Digital Repository: https://doi.org/10.5061/dryad.hv1kv (Halperin, Kalyuzhny, & Hawlena, 2017).
