# Supplementary material for: How to use (and not to use) movement‐based indices for quantifying foraging behaviour
Source: Methods Ecol Evol. 2017 Dec 18;9(4):1088–96. doi: 10.1111/2041-210X.12943 (PMC5993309; doi:10.1111/2041-210X.12943)
Supplement: Supplementary file 1 [file MEE3-9-1088-s001.docx]

**Bias in short samples of MPM**

**Figure S2**. Bias in short samples of MPM. For each of the 134 records of lizard behavior, which are equal or longer than 20 minutes, we calculated the MPM of the full time series. This was plotted against the mean of all possible 2 minutes samples of the long behavior (blue). In the lack of bias, this mean should be equal to the MPM of the full time series, hence falling on the red (X=Y) line. The deviation of the cloud of points from the red line therefore indicates the bias.
