# Supplementary material for: How to use (and not to use) movement‐based indices for quantifying foraging behaviour
Source: Methods Ecol Evol. 2017 Dec 18;9(4):1088–96. doi: 10.1111/2041-210X.12943 (PMC5993309; doi:10.1111/2041-210X.12943)
Supplement: Supplementary file 2 [file MEE3-9-1088-s002.docx]

# Appendix I - Determining the limits of the PTM-MPM plane:

Variables:

- MPM – Number of movements per minute [moves∙min^-1^] (calculated as in BOX I)
- PTM – The fraction of the time spent moving
- OD - Observation duration [min]
- M_min_ - The minimum duration for which a movement may be recognized [sec]
- S_min_ - The minimum duration for which a stop may be recognized [sec]
- MPM_max_ – The maximal MPM possible under the given M_min_ and S_min_ [moves∙min^-1^]
- PTM' – The PTM for which a maximal number of movements is possible given M_min_ and S_min_ (the PTM associated with MPM_max_)

Functions:

- remainder(x/y): the least positive remainder of the division x/y
- ceiling(x): a function which round a number up to the nearest integer
- floor(x): a function which round a number down to the nearest integer

Assumptions:

- OD>1 (at least one minute of observation)
- The numbers of movements and stops in an observation must be integers
- Total no. movements = (total no. stops) ± 1

Algorithm:

1. Calculating the "peak" of the triangle:

The maximum value of MPM for a given M_min_ and S_min_, MPM_max_, is equal to the number of times the segment M_min_-S_min_ may be repeated in the observation duration, rounded up or down to the nearest integer, and then divided by the observation duration.

The choice between rounding up or down depends on the remainder of the number of times the segment M_min_-S_min_ may be repeated in the observation duration.

If this remainder allows for another complete movement to occur:

$$\mathrm{remainder}\left( \frac{OD\cdot60}{M_{min}+ S_{min}} \right)\geq M_{min}$$

Then,

$${MPM}_{max}=\frac{\mathrm{ceiling}\left( \frac{OD\cdot60}{M_{min}+ S_{min}} \right)}{\mathrm{OD}}$$

In this case the number of movements may be maximized by assuming it is greater than the number of stops by 1.

But if the remainder does not allow for another complete movement to occur:

$\mathrm{remainder}\left( \frac{OD\cdot60}{M_{min}+ S_{min}} \right)< M_{min}$

Then,

$${MPM}_{max}=\frac{\mathrm{floor}\left( \frac{OD\cdot60}{M_{min}+ S_{min}} \right)}{\mathrm{OD}}$$

The PTM associated with MPM_max_, PTM', is equal to the total number of movements, multiplied by M_min_, and then divided by the observation duration.

$${PTM}^{'}=\frac{\left( {MPM}_{max}\cdot OD \right)\cdot M_{min}}{OD\cdot60}= \frac{{MPM}_{max}\cdot M_{min}}{60}$$

1. Calculating the maximal MPM associated with every PTM_i_ < PTM':

For low PTM values, which are smaller than PTM', the maximal number of movements per minutes, MPM_i_, is constrained by M_min_:

$${MPM}_{i}= \frac{\mathrm{floor}\left( \frac{{PTM}_{i}\cdot OD\cdot60}{M_{min}} \right)}{\mathrm{OD}}$$

We round down to the nearest integer in this case because a remainder does not allow for an extra movement (but does indicate that some movements were slightly longer than M_min_).

1. Calculating the maximal MPM associated with every PTM_i_ > PTM':

For high PTM values, which are higher than PTM', the maximal number of movements per minutes, MPM_i_, is constrained by S_min_:

$${MPM}_{i}=\frac{\mathrm{ceiling}\left( \frac{{(1-PTM}_{i})\cdot OD\cdot60}{S_{min}} \right)}{\mathrm{OD}}$$

We add one because the number of movements is maximized by assuming the number of movements exceeded the number of stops by 1 (it may never exceed the number of stops by more than 1).
