# Supplementary material for: How to use (and not to use) movement‐based indices for quantifying foraging behaviour
Source: Methods Ecol Evol. 2017 Dec 18;9(4):1088–96. doi: 10.1111/2041-210X.12943 (PMC5993309; doi:10.1111/2041-210X.12943)
Supplement: Supplementary file 3 [file MEE3-9-1088-s003.docx]

# Appendix II – supplementary methods

## Simulation model

To study the theoretical behavior of various statistics such as CPM and MPM, we used a simple model of movement (foraging or other). The model considers an individual that can be at one of two states: movement and rest. The individual does movements whose duration is 1 + M, when M is Poisson distributed, followed by stops whose duration is 1+ S where S is drawn from a Poisson. Despite its simplicity, this model can exemplify the constraints of the MPM-PTM space and the properties of the bias and error of MPM and CPM.

## Published data on species-level statistics

Data of the average measured values of PTM and MPM at the species level was collected from the literature. The data covers 162 species from a variety of families and phylogenetic background, including Agamidae, Sincidae, Lacertidae, Teiidae, Cordylidae, Polychrotidae, Phrynosomatidae, Tropiduridae, Corytophanidae, Leiocephalidae, Iguanidae, Opluridae, Chamaeleontidae, Crotaphytidae, Gymnophthalmidae, Gerrhosauridae, Eublepharidae, Anguidae, Hoplocercidae, Liolaemidae and Gekkonidae.

## Individual level data

We used behavioral sequences of 155 individual lizards that belong to six *Acanthodactylus* species *(A. aegyptius, A. scutellatus, A. boskianus, A. beershebensis, A. schreiberi and A. schmidti)* and to *Podarcis lilfordi*, measured in 6 different field sites in Israel, Jordan and Spain. All observations were conducted at around the peak of foraging daily activity to control for variation in lizard movement pattern resulting from non-foraging behaviors (e.g., thermoregulation, Perry 2007). Each lizard was located by random search and then observed from a distance of approximately 4 m. The average observation duration was 20.5min ± 1.7 SD, after deleting the first 3 min of every observation to decrease variation resulting from transient behaviors before habituation of the lizard to the observer. The behavior was recorded on a palm-top with the software FIT (Held and Manser 2005). Observations were conducted on hatchling, juveniles and adults. Only lizards with intact tails were recorded to decrease variability associated with changes in activity that may result from tail autotomy (Martin and Salvador 1997). Observation on lizards that were engaged in thermoregulation (basking or transient movements between shaded spots) or lizards that showed a strong response to the observer (i.e., escape behavior and intensive refuge use) were not included. We used *Mmin* of approximately 4sec *and Smin* of approximately 3 sec in all observations.

**References**

Held J, Manser T (2005) A PDA-based system for online recording and analysis of concurrent events in complex behavioral processes. Behavior Research Methods, 7,155–164.

Martin J, Salvador A (1997) Effects of tail loss on the time-budgets, movements, and spacing patterns of Iberian rock lizards, *Lacerta monticola*. Herpetologica, 53,117–125.

Perry G (2007) Movement patterns in lizards: measurement, modality, and behavioral correlates. In: Reilly M, McBrayer LD, Miles DB eds). Lizard ecology: the evolutionary consequences of foraging mode. Cambridge University Press, Cambridge, pp 13–48.
