# Supplementary material for: How to use (and not to use) movement‐based indices for quantifying foraging behaviour
Source: Methods Ecol Evol. 2017 Dec 18;9(4):1088–96. doi: 10.1111/2041-210X.12943 (PMC5993309; doi:10.1111/2041-210X.12943)
Supplement: Supplementary file 4 [file MEE3-9-1088-s004.docx]

# Appendix III: Bias in MPM

Here we study the causes and properties of the bias in the naïve estimator of MPM, $\hat{MPM}$, which is calculated simply by dividing the observed number of movements by the observation duration, *OD*. We further suggest an unbiased estimator and derive a bias-correction formula for published $\hat{MPM}$ values. We start by showing a simple example, and then derive the general formula for the bias in MPM and the bias-correction formula.

As mentioned in the main text, the cause of the bias in MPM is the “gain” of moves in the initial and terminal parts of sampling units, because each time a sampling unit begins or ends with a move, a whole move is counted, while only a fraction is actually observed. This is illustrated in Figure S1, which considers a simple, discrete time hypothetical example of an animal whose movement consists of three seconds of movement, followed by two seconds of rest. For this example we assume that the sampling unit is of eleven seconds, and all five possible distinct sampling units are presented (The sampling unit that begins in the sixth second, after sampling unit E, is identical to sampling unit A, and so forth) along with the number of moves that are counted and the true number of moves that is actually observed. Assuming that the sampling begins and ends at a random time, the probability for all five possible sampling units is 0.2, hence the mean number of counted moves is 2.6 and after division by OD (=11/60), $\hat{MPM}=14.18$. The true MPM here is 12, as 12 five second sections of move and stop occur in each minute on average on the long run, and we would have got this estimate had we used the true observed number of moves (2.2). Generally, the total gain is a result of gain in the beginning of the sampling unit (initial gain) and at the end of the sampling unit (terminal gain), which together sum to the difference between the counted moves and the observed moves.


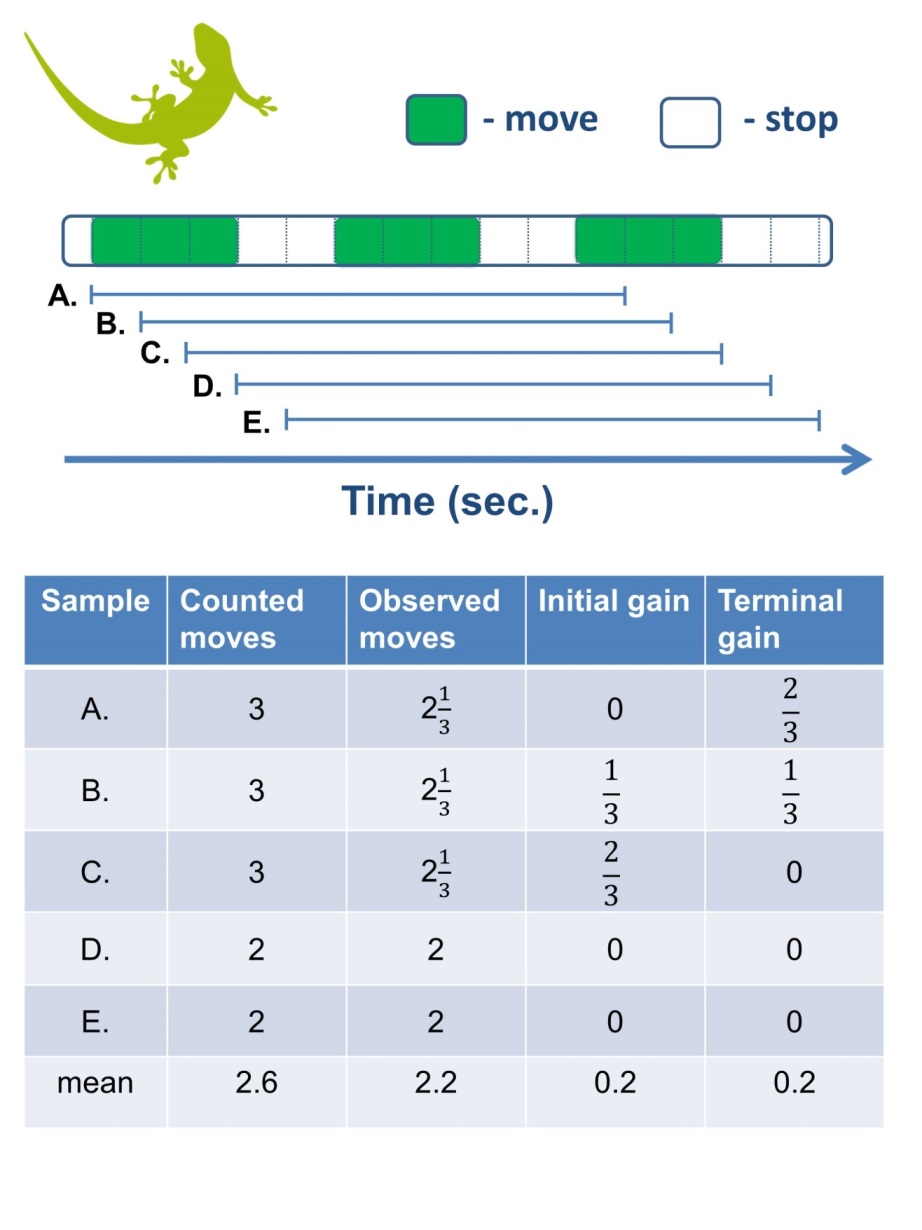


**Figure S1 -** Simple numerical example of the $\hat{MPM}$causes of bias. Each dashed segment represents one second, and since all moves last three seconds and all stops last two, the lines A-E represent the 5 possible sampling units of 11 seconds. The table compares the counted number of moves in each sampling unit and the number of moves that were actually observed. The difference among the two is a result of the gain of moves in the initial and terminal parts of the sampling unit.

This example can be generalized to a situation in which moves are of different lengths, and the probability that a move is of length *m* is P(*m*). For the sake of generality and simplicity we will study the bias in continuous time. Our analyses showed that the effect of discretizing time is negligible. Assuming that there is no temporal correlation structure in the moves and stops (each move and stop lengths are independent of the previous moves and stops) and that sampling begins and ends at a random time in a long sequence, then the expected number of moves that are “gained” on one edge (initial or terminal) by counting fractions of moves as whole moves, E(*gain*), is

(1) $E\left( gain \right)=\int E\left( gain|\mu\right)P(\mu)dm$

where *μ* is an event when the edge is contained within a move of length *m*.

Given *μ*, and since we assumed that the sampling begins and ends at random times, on average we will “cut” the move in half. Therefore, the expected number of moves gained give *μ* is simply 0.5.

The probability that the edge of the sampling unit would be contained in a move of length *m* (and not in a stop) is:

(2) $P\left( \mu\right)=\frac{mP(m)}{AM+AS}$ ,

where *AM* and *AS* are the average duration of moves and stops, respectively. This is easily understood if we remember that the sum of this expression over all *m* is the probability that the edge would be contained in a move of any length, which equals *AM*/(*AM*+*AS*). This sum is simply PTM (asymptotically for long observations).

Substituting equations (2) into (1) gives:

(3) $E\left( gain \right)=0.5\int\frac{mP(m)}{AM+AS}dm=\frac{AM}{2(AM+AS)}=\frac{PTM}{2}$.

Multiplying this expression by two (since there are two edged) and dividing the total gain along the entire observation duration gives the expression for the bias in MPM:

(4) $Bias=E\left( \hat{MPM}-MPM \right)=\frac{PTM}{OD}$.

This prediction is tested in the main text.

In Figure 3 of the main text we examined the relative bias, which is obtained by dividing both sides by MPM, and remembering that (asymptotically for long observations) MPM=1/(*AM*+*AS*):

(5) ${Bias}_{rel}=\frac{E\left( \hat{MPM}-MPM \right)}{MPM}=\frac{AM}{OD}$.

Figure 3 in the main text shows using simulations that, as predicted by this analysis, this value is independent of AS and linearly dependent on *AM* and on 1/*OD*. Hence, the bias will be particularly substantial for short observation of animals with long move durations or PTM. An important feature of this result is that, unlike many classic situations, the bias of this estimator is independent of sample size, making it inconsistent.

From eq. 4 it is evident that the bias corrected estimator for MPM, MPM’, should be:

(6) $MPM^{'}=\hat{MPM}-\frac{PTM}{OD}$,

The aforementioned derivation of *MPM'* has been done at the level of an individual sample and requires data on $\hat{MPM}$, *PTM* and *OD* for every sample. Most data reported in the literature include only the average value of $\hat{MPM}$, *PTM* and (sometimes) *OD* for an entire species. Therefore we analyzed the application of *MPM'* for species averages. We would like to know the mean of *MPM'*, E(*MPM'*), calculated on multiple individuals, since the correction is valid at that level.

Taking expectations on both sides:

(7) $E\left( MPM^{'} \right)=E\left( \hat{MPM} \right)-E\left( PTM \right)E\left( \frac{1}{OD} \right)=E\left( \hat{MPM} \right)-\frac{60E(PTM)}{H(OD)}$,

where H(*OD*) is the harmonic mean of *OD*. This step is based on the assumption that measurements of *PTM* are independent on *OD* (since it is unbiased).

First, if the durations of observations are constant, there is no problem to evaluate $E\left( MPM^{'} \right)$ by using the reported averages of $\hat{MPM}$ and *PTM*. However, in the likely case when there is variability in *OD*, and only the (arithmetic) average of observation time is reported, we can only calculate a different measure, *MPM''*:

(8) $MPM^{''}=\bar{\left( \hat{MPM} \right)}-\frac{\bar{PTM}}{\bar{OD}}$, where the overbar denotes an (arithmetic) average.

Hence, the full bias correction involves reducing a number proportional to the reciprocal of the harmonic mean, while the feasible correction, *MPM''*, involved reducing a number proportional to the reciprocal of the arithmetic mean. Since the harmonic mean is never larger than the arithmetic mean, we will always correct some (or all) the bias, but never create extra bias. Our numerical simulations confirmed this result. We can therefore recommend using the *MPM'* correction even when only species-level averages are available.

Since we made a few assumptions in this deviation, and particularly that there is no correlation between the duration of a move or stop and other moves and stops, and that the duration of the observation is long (to transform *AM*/(*AM*+*AS*) to *PTM*), we recommend using this expression only as a correction of published results (that usually report observation duration and PTM). If raw data on moves and stops is available, we recommend avoiding the problem altogether by using an estimator that inherently does not cut moves and stops in the middle – the number of changes per minute (*CPM*). This statistic is calculated simply by dividing the number of events when the animal initiated moving or stopped (changes) by the observation duration – 1 second (or other minimal time unit to which the observations were discretized, since no change can be observed in the last time unit of the observation). As shown in figures 3-4 of the main text, this statistic is unbiased and may even have lower variance than *MPM*.
