# Supplementary material for: How to use (and not to use) movement‐based indices for quantifying foraging behaviour
Source: Methods Ecol Evol. 2017 Dec 18;9(4):1088–96. doi: 10.1111/2041-210X.12943 (PMC5993309; doi:10.1111/2041-210X.12943)
Supplement: Supplementary file 6 [file MEE3-9-1088-s006.docx]

**References for Appendix IV**

Anderson, R.A. (1993). An analysis of foraging in the lizard, Cnemidophoms tigris. Biology of Whiptail Lizards (eds J.W. Wright & L.J. Vitt), pp. 83–116. Oklahoma Museum of Natural History, Norman.

Anderson, R.A. & Karasov, W.H. (1981). Contrasts in energy intake and expenditure in sit-and-wait and widely foraging lizards. Oecologia, 49, 67–72.

Andrews, R.M. (1979). Evolution of life histories: A comparison of anolis lizards from matched island and mainland habitats. Breviora., 454, 1–51.

Butler, M.A. (2005). Foraging mode of the chameleon, Bradypodion pumilum: A challenge to the sit-and-wait versus active forager paradigm? Biological Journal of the Linnean Society, 84, 797–808.

Cooper, W.E. (2005). Duration of movement as a lizard foraging movement variable. Herpetologica, 61, 363–372.

Cooper, W.E., Castañeda, G., la Peña, C.G. De, Wilson, B., Caldwell, J.P. & Vitt, L.J. (2013). Foraging modes of some Jamaican, Costa Rican, and Mexican lizards. Herpetology Notes, 6, 591–597.

Cooper, W.E., Pérez-Mellado, V. & Hawlena, D. (2014). Foraging by the omnivorous lizard Podarcis lilfordi: Effects of nectivory in an ancestrally insectivorous active forager. Journal of Herpetology, 48, 203–209.

Cooper, W.E., Vitt, L.J., Caldwell, J.P. & Fox, S.F. (2001). Foraging modes of some American lizards: Relationships among measurement variables and discreteness of modes. Herpetologica, 57, 65–76.

Cooper, W.E., Vitt, L.J., Caldwell, J.P. & Fox, S.F. (2005). Relationships among foraging variables, phylogeny, and foraging modes, with new data for nine North American lizard species. Herpetologica, 61, 250–259.

Cooper, W.E. & Whiting, M.J. (2000). Ambush and active foraging modes both occur in the scincid genus Mabuya. Copeia, 2000, 112–118.

Cooper, W.E. & Whiting, M.J. (1999). Foraging modes in lacertid lizards from southern Africa. Amphibia-Reptilia, 20, 299–311.

Cooper, W.E., Whiting, M.J. & Van Wyk, J.H. (1997). Foraging modes of cordyliform lizards. South African Journal of Zoology, 32, 9–13.

Cooper, W.E., Whiting, M.J., Van Wyk, J.H. & Mouton, P.L.F.N. (1999). Movement- and attack-based indices of foraging mode and ambush foraging in some gekkonid and agamine lizards from southern Africa. Amphibia-Reptilia, 20, 391–399.

Eifler, D.A. & Eifler, M.A. (1999). Foraging behaviour and spacing patterns of the lizard *Oligosoma grande*. Journal of Herpetology, 33, 632–639.

Ellinger, N., Schlatte, G., Jerome, N. & Hödl, W. (2001). Habitat use and activity patterns of the neotropical arboreal lizard *Tropidurus* (= Uracentron) *azureus werneri* (Tropiduridae). Journal of Herpetology, 35, 395–402.

Gasnier, T.R., Magnusson, W.E. & Lima, A.P. (1994). Foraging activity and diet of four sympatric lizard species in a tropical rainforest. Journal of Herpetology, 28, 187–192.

Greeff, J.M. & Whiting, M.J. (2000). Foraging-mode plasticity in the lizard *Platysaurus broadleyi*. Herpetologica, 56, 402–407.

Hawlena, D. (2009). Colorful tails fade when lizards adopt less risky behaviors. Behavioral Ecology and Sociobiology, 64, 205–213.

Huey, R.B. & Pianka, E.R. (1981). Ecological consequences of foraging mode. Ecology, 62, 991–999.

Husak, J.F. & Ackland, E.N. (2003). Foraging mode of the reticulate collared lizard, *Crotaphytus reticulatus*. The Southwestern Naturalist, 48, 282–286.

Irschick, D.J. (2000). Comparative and behavioral analyses of preferred speed: Anolis lizards as a model system. Physiological and Biochemical Zoology, 73, 428–437.

Karasov, W.H. & Anderson, R.A. (1984). Interhabitat differences in energy adquisition and expendiature in a lizard. Ecology, 65, 235–247.

Kirchhof, S., Linden, J., Rödder, D. & Richter, K. (2010). Foraging mode *of Australolacerta rupicola* (FitzSimons, 1933)(Sauria: Lacertidae): Evidence of seasonal variation in an extremely active predator? Journal of Natural History, 44, 2941–2953.

Magnusson, W.E., Paiva, L.J. De, Moreira, R., Franke, R., Kasper, L.A., Lima, A.P., Rocha, D. A & Franke, C.R. (1985). The correlates of foraging mode in a community of Brazilian lizards. Herpetologica, 41, 324–332.

McConnachie, S. & Whiting, M. (2003). Costs associated with tail autotomy in an ambush foraging lizard, *Cordylus melanotus melanotus*. African Zoology, 38, 57–65.

Menezes, V.A., Amaral, V.C., Sluys, M. V. & Rocha, C.F.D. (2006). Diet and foraging of the endemic lizard *Cnemidophorus littoralis* (Squamata, Teiidae) in the restinga de Jurubatiba, Macaé, RJ. Brazilian journal of biology, 66, 803–807.

Moermond, T. (1979). The influence of habitat structure on Anolis foraging behavior. Behaviour, 70, 147–167.

Mori, a. & Randriamahazo, H.J. a R. (2002). Foraging mode of a Madagascan iguanian lizard, *Oplurus cuvieri cuvieri*. African Journal of Ecology, 40, 61–64.

Mouton, P.L.F.N., Geertsema, H. & Visagie, L. (2000). Foraging mode of a group-living lizard, *Cordylus cataphractus* (Cordylidae). African Zoology, 35, 1–7.

Murray, I.W., Fuller, A., Lease, H.M., Mitchell, D., Wolf, B.O. & Hetem, R.S. (2014). The actively foraging desert lizard *Pedioplanis husabensis* (Husab Sand Lizard) behaviorally optimizes its energetic economy. Canadian Journal of Zoology, 92, 905–913.

Nemes, S. (2002). Foraging mode of the sand lizard, Lacerta agilis, at the beginning of its yearly activity period. Russian Journal of Herpetology, 9, 57–62.

Paulissen, M.A. (2001). Ecology and behavior of lizards of the parthenogenetic *Cnemidophorus laredoensis* complex and their gonochoristic relative *Cnemidophorus gularis*: Implications for coexistence. Journal of Herpetology, 35, 282–292.

Perry, G. (2007). Movement patterns in lizards: Measurement, modality, and behavioral correlates. Lizard Ecology: The Evolutionary Consequences of Foraging Mode (ed D.B. Reilly, Stephen M.; McBrayer, Lance B.; Miles), pp. 13–48. Cambridge University Press, New York.

Perry, G. (1999). The evolution of search modes: Ecological versus phylogenetic perspectives. The American Naturalist, 153, 98–109.

Perry, G. & Buden, D.W. (1999). Ecology, behavior and color variation of the green tree skink, *Lamprolepis smaragdina* (Lacertilia: Scincidae), in Micronesia. Micronesia, 31, 263–273.

Pianka, E.R., Huey, R.B. & Lawlor, L.R. (1979). Niche segregation in desert lizards. Analysis of ecological systems (eds D.J. Horn, R. Mitchell & G.R. Stairs), pp. 67–115. Ohio State University Press, Columbus.

du Plessis, I.J. & Mouton, P.L.F.N. (2012). Foraging strategies of coexisting lacertid lizards in the arid Tankwa Karoo basin of South Africa. African Zoology, 47, 113–118.

Reaney, L.T. & Whiting, M.J. (2002). Life on a limb: Ecology of the tree agama (Acanthocercus a. atricollis) in southern Africa. Journal of Zoology, 257, 439–448.

Rensburg, D.A.J. van, Mouton, P. le F.N. & van Niekerk, A. (2009). Why cordylid lizards are black at the south-western tip of Africa. Journal of Zoology, 278, 333–341.

Sales, R.F.D. & Freire, E.M.X. (2015). Diet and foraging behavior of *Ameivula ocellifera* (Squamata: Teiidae) in the Brazilian semiarid Caatinga. Journal of Herpetology, 49, 579–585.

Scales, J.A., King, A.A. & Butler, M.A. (2009). Running for your life or running for your dinner: What drives fiber-type evolution in lizard locomotor muscles? The American naturalist, 173, 543–553.

Shaffer Jr., D.T. & Whitford, W.G. (1981). Behavioral responses of a predator, the round-tailed horned lizard, *Phrynosoma modestum* and its prey, Honey Pot Ants, Myrmecocystus spp. American Midland Naturalist, 105, 209–216.

Taylor, J.A. (1986). Food and foraging behaviour of the lizard, Ctenotus taeniolatus. Australian Journal of Ecology, 11, 49–54.

Du Toit, D. a., Mouton, P.L.F.N., Geertsema, H. & Flemming, A.F. (2002). Foraging mode of serpentiform, grass-living cordylid lizards: a case study of *Cordylus agnuina*. African Zoology, 37, 141–149.

Verwaijen, D. & Van Damme, R. (2007a). Correlated evolution of thermal characteristics and foraging strategy in lacertid lizards. Journal of Thermal Biology, 32, 388–395.

Verwaijen, D. & Van Damme, R. (2007b). Does foraging mode mould morphology in Lacertid lizards? Journal of Evolutionary Biology, 20, 1950–1961.

Verwaijen, D. & Van Damme, R. (2008a). Foraging mode and its flexibility in Lacertid lizards from Europe. Journal of Herpetology, 42, 124–133.

Verwaijen, D. & Van Damme, R. (2008b). Wide home ranges for widely foraging lizards. Zoology, 111, 37–47.

Vitt, L.J., Souza, R.A., Sartorius, S.S., Avila-Pires, T.C.S. & Espósito, M.C. (2000). Comparative ecology of sympatric gonatodes (Squamata: Gekkonidae) in the western Amazon of Brazil. Copeia, 1, 83–95.

Vitt, L.J., Zani, P.A., Caldwell, J.P. & Carrillo, E.O. (1995a). Ecology of the lizard *Kentropyx pelviceps* (Sauria: Teiidae) in lowland forest of Ecuador. Canadian Journal of Zoology, 73, 691–703.

Vitt, L.J., Zani, P.A. & Durtsche, R.D. (1995b). Ecology of the lizard *Norops oxylophus* (Polychrotidae) in lowland forest of southeastern Nicaragua. Canadian Journal of Zoology-Revue Canadienne De Zoologie, 73, 1918–1927.

Vitt, L., Zani, P. & Lima, A. (1997). Heliotherms in tropical rain forest: The ecology of *Kentropyx calcarata* (Teiidae) and *Mabuya nigropunctata* (Scincidae) in the Curua-Una of Brazil. Journal of Tropical Ecology, 13, 199–220.

Wasiolka, B. (2007). The Impact of overgrazing on reptile diversity and population dynamics of *Pedioplanis l. Lineoocellata* in the southern Kalahari. PhD Thesis University of Potsdam.

Werner, Y.L. & Chou, L.M. (2002). Observations on the ecology of the arrhythmic equatorial gecko *Cnemaspsis kendallii* in Singapore. The Raffles Bulletin of Zoology, 50, 185–196.

Werner, Y.L., Okada, S., Ota, H., Perry, G. & TokunagaShoji. (1997). Varied and fluctuating foraging modes in nocturnal lizards of the family Gekkonidae. Asiatic Herpetological Research, 7, 153–165.

Wymann, M.N. & Whiting, M.J. (2002). Foraging ecology of rainbow skinks (*Mabuya margaritifer*) in southern Africa. Copeia, 4, 943–957.

Zero, V.H., Eifler, D.A. & Powell, R. (2009). Foraging behavior of the lizard *Ameiva erythrocephala* Daudin, 1802 (Squamata: Sauria: Teiidae). Herpetozoa, 22, 167–171.
